# Use of Augmented Reality as a Tool to Support Cargo Handling Operations at the CARGO Air Terminal

**DOI:** 10.3390/s24041099

**Published:** 2024-02-08

**Authors:** Agnieszka A. Tubis, Anna Jodejko-Pietruczuk, Tomasz Nowakowski

**Affiliations:** Department of Technical Systems Operation and Maintenance, Faculty of Mechanical Engineering, Wroclaw University of Science and Technology, Wyspianskiego Street 27, 50-370 Wroclaw, Poland; anna.jodejko@pwr.edu.pl (A.J.-P.); tomasz.nowakowski@pwr.edu.pl (T.N.)

**Keywords:** AR technology, air transport, automatization, information flow, logistic processes

## Abstract

(1) Background: A current trend observed in the logistics sector is the use of Industry 4.0 tools to improve and enhance the efficiency of cargo handling processes. One of the popular solutions is an augmented reality system that supports operators in everyday tasks. The article aims to present design assumptions for implementing an augmented reality system to support air cargo handling at the warehouse. (2) Methods: Research was carried out based on a five-stage analytical procedure, aiming to analyze the current state and identify the potential for implementing the AR system. The following methods were used to collect data: co-participant observations, process analysis, direct interviews, analysis of internal documentation, and applicable legal regulations. (3) Results: The conducted research allowed for identifying information flows accompanying cargo flows and developing a project to automate selected information flows. The obtained results made it possible to identify operations for which the AR system’s implementation will increase their effectiveness and efficiency. (4) Conclusions: The obtained results identified the need to develop a hybrid algorithm for arranging cargo in the warehouse and to build a system supporting self-verification of markings on air cargo.

## 1. Introduction

A current trend observed in the logistics sector is the use of Industry 4.0 tools to improve and enhance the efficiency of cargo handling processes. This trend, referred to as Logistics 4.0, is now an integral part of the development of the fourth industrial revolution. Logistics 4.0 uses new technologies to support the operation of traditional logistics systems [1]. Implemented cyber–physical solutions allow for improved cargo handling and introduce automation of storage system transportation and decentralized software control [2]. At the same time, the digitization of logistics processes enables companies to monitor material flows in real time and better handle handling units [3,4].

The importance of the Logistics 4.0 concept is growing, as evidenced by the steadily increasing number of publications in this area [5]. An important element of it is the digitization of warehouse-related processes. This is because warehousing is one of the primary logistics processes and is an essential part of the integration of all operations in the supply chain. As Hamdy et al. [6] point out, this process is a crucial part of product flows due to its involvement in achieving optimum and continuous operation of the production and distribution processes. For this reason, the digital solutions of Industry 4.0 are finding numerous applications in warehouse processes, and their implementation makes it possible to create smart warehouses, called Warehouses 4.0. According to the research presented in [7], the following solutions are most commonly used in these facilities: augmented reality (AR), RFID, Internet of Things, visual technology, and automated storage systems. Characteristics of storage facilities based on these solutions include, in particular, interoperability, virtualization, decentralization, real-time aspects, service orientation, modularity, and reconfigurability [8].

The implementation of improvements using Industry 4.0 solutions assumes particular importance in the case of handling cargo moved in transportation systems. In this handling, fast and precise identification of a shipment, real-time monitoring of its flow, coordinated operations performed in a fixed order according to an established procedure, and accurate information delivered to the operator in a readable form are of particular importance. In addition, in the case of air cargo handling, a critical element is the maintenance of safety procedures, short time slots for handling operations, and identification of markings for specific types of shipments that determine the application of specific handling procedures. For this reason, CARGO air cargo handling terminals are reporting a need for dedicated digital solutions to eliminate human error, reduce turnaround times for handling operations, and increase service efficiency.

The article’s purpose is to present the design of functionality for an augmented reality system that will support the storage handling of air cargo. The published results are part of the research conducted under the project “A virtual support system for Cargo handling processes at airports, based on augmented reality technologies”. The research was conducted in cooperation with a selected airport in Poland, which handles cargo in domestic and international distribution. The results presented in the article allow us to formulate the following main contributions:

- Identification of information flows related to air cargo handling at the CARGO terminal, along with identification of potential for automation.
- Formulation of the research procedure stages for the design of AR tools supporting air cargo handling.
- Presentation of the functionality design of an AR tool to support warehouse operators.
- Identification of challenges associated with the application of AR technology in air cargo handling.

**Figure 1:**
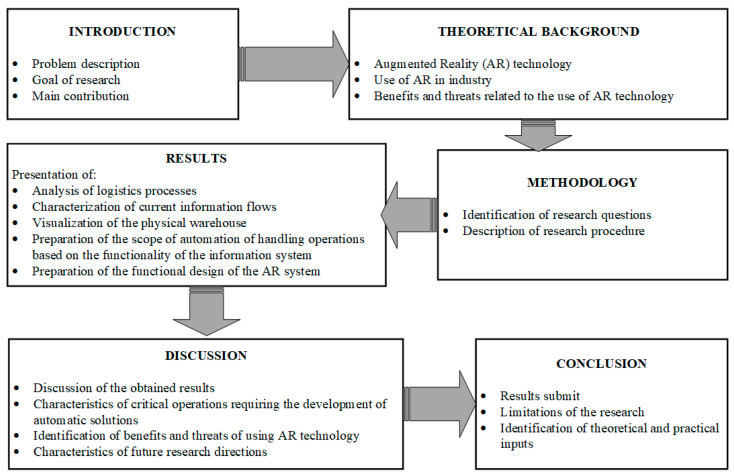
Structure of the article.

Figure 1 shows the adopted structure of the article.

## 2. Theoretical Background

Augmented reality technology is one of the primary tools of Industry 4.0, the application of which is described in the areas of manufacturing [9], maintenance [10], and internal logistics systems [7]. AR technology allows a person to see a computer-generated virtual world that is simultaneously integrated with the real world. Thus, it can be said that AR tools can be used as an interface providing a link between digital information and the physical world [11]. Van Krevelan and Poelman went a step further in their definition, stating that AR “is an emerging technology with which a person can see more than others see, hear more than others hear, and perhaps even touch, smell and taste things that others cannot [12]”. Daponte et al. [13], on the other hand, proposed a measurement approach to the definition of AR, defining it as a technology that enriches the user’s sensory perception by showing information about the surrounding environment (e.g., physical quantities) that cannot be perceived with the five senses.

AR is a part of mixed reality [12]. That is, it is a form that combines real and virtual environments. Azuma et al. [14] pointed out three primary characteristics of an AR system: (a) it combines real-world and virtual objects, (b) it runs in real time, and (c) it allows interaction between users and virtual objects. The basic structure of an AR system consists of four elements [13]:

- A video camera that transmits an actual image of the environment in which the user is located.
- Tracking Module, which monitors the relative position and orientation of the camera in real time. This module can be based on every type of sensor technology: (1) 9D IMU (3-axis accelerometer, 3-axis gyroscope, and 3-axis magnetometer), (2) ultrasonic sensors, (3) video cameras, (4) GPS modules, and (5) RFID devices.
- Graphic Processing Module processes images captured by the video camera and adds virtual objects to them.
- Display provides users with an integrated image of the physical world combined with virtual objects.

Four visualization technologies are available on the market for AR systems [15]: head-mounted displays (HMDs), handheld devices (HHDs), static screens, and projectors. These systems can be stationary or mobile devices depending on the defined visualization task. The research results presented in [16] indicate that HMDs are the most applicable. Also, Masood et al.’s [15] research indicates that HMDs are the focus of research work and solutions implemented in industry. This is mainly due to the fact that their use does not restrict the operator’s movement but instead allows hands-free access and reading of information, which significantly speeds up operations [17]. Nowadays, AR smart glasses (ARSGs) are increasingly being used, showing good potential for industrial applications [18]. ARSGs support the operator’s work as they are equipped with numerous functions—from displaying information to tracking, distributing, and storing data about the surrounding environment and the user [19]. It is worth noting, however, that research results show (among others [16]) that most AR deployments occur on various devices. This is because many implementations render in a fixed computer and synchronize the real and virtual objects in a mobile device [16]. It should also be noted that creating an AR system from scratch is difficult and time-consuming. For this reason, several frameworks and platforms are available on the market that allow developers to focus on higher-level applications rather than low-level implementations. An example collection of such platforms is presented in [20].

Research by de Souza Cardoso et al. [16] shows that the primary areas of application of AR technology in industry are manual assembly, robot programming and operations, maintenance, process monitoring, training, process simulation, quality inspection, picking process, operational setup ergonomics, and safety. AR is therefore primarily used in processes for which it is possible to increase productivity by

- Providing flexible, hands-free, and real-time information delivery [21,22];
- Reducing the incidence of human error (e.g., incorrect picking, assembly, or maintenance) [23].

At the same time, the results show that most of the described applications did not consider the specifics of a particular sector but were concerned with supporting general industrial processes. There are, of course, AR solutions dedicated to the specifics of a particular industry. According to Cardoso et al.’s research, these usually apply to architecture, engineering, construction and facilities management (AEC/FM), aeronautics, automotives, electronics/automation, energy, government, logistics, marine, or mechanical aspects [16].

In logistics, the leading application area for AR is the picking process [24]. In this process, AR tools typically support the operator in reaching the right location and indicating the quantity to be picked [21,25,26]. Research indicates that using augmented reality to communicate order completion information improves time efficiency and picking accuracy (among others [27]). Stoltz et al. [28] prove in their research that the three other key warehouse processes also have great potential regarding use of AR. Although publications on the implementation of AR in receiving, storing, and shipping processes are currently very limited, according to Stoltz et al. [28], implementing this technology, in their case, will provide similar effects to those observed in the picking process.

Several publications have appeared in recent years to review the literature focused on using AR technology in logistics. Examples of such publications include [29,30]. These reviews argue that the main benefit of using AR in warehouse operations is primarily to increase visualization and product identification. Rejeb et al., based on their results, identified the main benefits of improving visualization and identification in warehouse processes [30]:

- Increased visual control and monitoring of products and stock-keeping units;
- Efficiency and potential cost savings through minimization of errors during product identification, losses, and damages;
- Increased efficiency and productivity through minimization of search time, mis-picks, fatigue, and errors;
- Efficient inventory management and order-picking processes through better visualization and guidance.

In addition, ref. [29] presented the benefits of using ARSGs in warehouse operations. The results of the analyses have made it possible to identify four areas where smart glasses add value to logistics processes. These include improvements in the following:

- Visualization: (a) the information displayed in the operators’ field of view decreases task completion times by eliminating unnecessary head and body movement; (b) documenting all operator actions, continuous monitoring, naturally following the user’s attention, providing more information;
- Interaction: (a) building safer and more productive work environments by promoting beneficial uses of technology for people; (b) being able to identify and mitigate risks with ARSGs; (c) improving human–environment interaction and human perception to complete tasks;
- User convenience: (a) efficient, versatile, and comfortable to wear; (b) does not distract workers; (c) enables hands-free access to information;
- Navigation: (a) ability to move quickly along optimized paths; (b) precise location and the ability to track position at all times; (c) easily find physical targets.

Several publications also present challenges and risks associated with implementing AR technology. According to the study in [29], many articles group these risks into three categories: technical, organizational, and ergonomic aspects. A more elaborate classification of challenges and constraints was formulated by de Souza Cardoso et al. [16], according to which there are five categories: users’ health and acceptance; tracking methods; projection quality, accuracy, and interaction; hardware; and development complexity. Also noteworthy is the barrier classification presented by Stoltz et al. [28], who distinguish hardware limitations, software challenges, acceptance, and cost.

The literature review confirms that our research and the defined cognitive goal align with the current research trends. In turn, the adopted specification of the developed tool and its adaptation to the needs of air cargo handling processes fill the current research gap related to the need for more publications on adapting AR systems to the specifics of the studied sector.

## 3. Methods

The research team’s task was to visualize the actual system handling cargo flow within the CARGO air terminal and identify logistics operations that can be supported by information technology, including AR technology. During the research, team members worked closely with air terminal employees from all levels of the organization—operators, team leaders, managers, and management. The research approach adopted made it possible to gather information on logistics processes, which were verified at different levels of management. As a result, the mapped processes and developed system recommendations considered the perspective of the operational position and the management of the entire supply chain.

**Figure 2:**
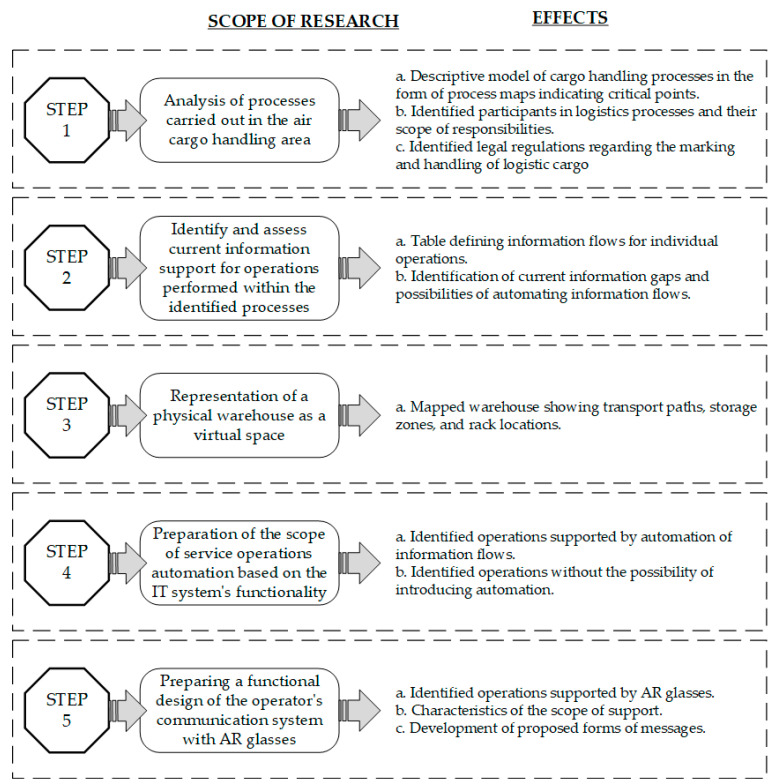
Research procedure.

The research procedure included five stages, which are shown in Figure 2. The research procedure was conducted to answer the following questions:

- RQ1: How is the air cargo handling process carried out at the CARGO terminal, what factors regulate its execution, and what information flows accompany the logistics operations performed?
- RQ2: Which information flows can be automated using an appropriate information system?
- RQ3: Which handling operations can be supported by augmented reality solutions, and how will this affect process execution?

For each step, the expected sub-results were formulated, the achievement of which was critical to achieving the formulated research objective.

Identification of the current model of the process information system, followed by its analysis from the point of view of the flows and form of transmitted information, were necessary to develop a system of support for the operator by AR technology in the process of cargo handling. Based on the process analysis, the main processes were identified in which the coordination of individual operations should be supported by the information system being developed. Organizational requirements and applicable operating procedures were determined for these processes based on training documentation for warehouse workers. Process maps and organizational requirements were established based on observations accompanying warehouse employees, face-to-face structured interviews conducted among team leaders and managers, and internal documentation of the CARGO terminal and applicable legislation.

Subsequently, the current level of information support for the various operations of the analyzed processes was identified and evaluated. Accompanying observations, direct interviews with employees and analysis of internal documentation were used to identify information flows. The scope of the identification carried out made it possible to create a knowledge base of information flows accompanying logistics operations and included

- Information feeding the implementation of operations;
- The current form of obtaining in-feed information;
- The person carrying out operations at the terminal;
- The type of authorization required to carry out the operation;
- Information generated as a result of the execution of the operation;
- The current form of the generated output information.

All identified feed information should be evaluated as necessary to implement the operation under analysis correctly. Thus, based on the compilation created, it was possible to identify the information gaps present and the potential for automating the process of information flow that accompanies cargo handling operations.

The conducted process analysis was the basis for designing the functionality of the information system, including the operator’s communication with AR glasses. However, to ensure the system’s required functionality, it is necessary to supplement the process map with a visualized physical object of the warehouse. The task of the information system is to support employees in managing the cargo flow in the warehouse area. Therefore, it is necessary to feed it with knowledge of available warehouse locations, dimensions, and locations in defined service zones. For the AR tool being created, the transport paths operators will use must also be mapped in the system. This necessitates the creation of virtual mapping of the warehouse with the marking of important points that are critical from the point of view of the moving operators and the loads handled.

In the following research stage, operations were specified for each of the processes identified in Stage 1, for which an automated cargo flow support system could be introduced. The scope of automation was determined for the highlighted operations, and the characteristics of the improvement to be introduced were prepared. The scope of automation was determined based on (1) the identified functionality of the WMS system, (2) potential opportunities identified in the analysis of similar market solutions, and (3) the identified demand resulting from the implementation of the AR tool. In addition, face-to-face interviews were conducted with experienced warehouse operators, based on which their information needs in handling operations were identified.

The following research stage focused primarily on developing guidelines for communication between the operator and AR glasses. To this end, critical operations (from the point of view of process continuity) and complicated operations (especially for a new employee) were identified, the implementation of which could be supported by AR technology. The extent of this support was determined based on the operations’ characteristics and the demand reported by operators in the face-to-face interviews conducted. On this basis, the proposed functions of the AR system were formulated, along with their characteristics and the proposed form of messages.

## 4. Results

The project aims to develop a system to support air cargo handling using augmented reality technology. The developed AR system is to correspond to the specifics of the logistics processes carried out at air cargo terminals and the security requirements of air transport. Therefore, the first research stage concerning formulating assumptions for the created information system and AR-supporting cargo handling processes required testing in a real environment. For this purpose, a regional CARGO terminal handling air cargo in domestic and international transport was selected for the study. The terminal offers comprehensive air freight service, including special shipments, cargo security checks, and customs handling. However, it should be noted that the results obtained additionally included consultation with experts from other airports to ensure the conclusions’ universality.

### 4.1. Stage 1—Analysis of Logistics Processes

**Figure 3:**
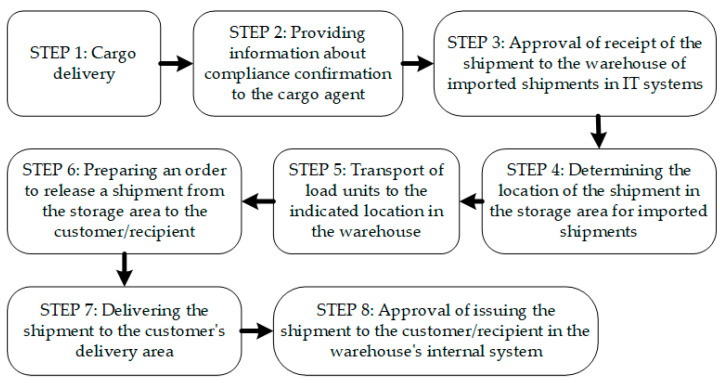
Sub-processes for handling imported shipments.

**Figure 4:**
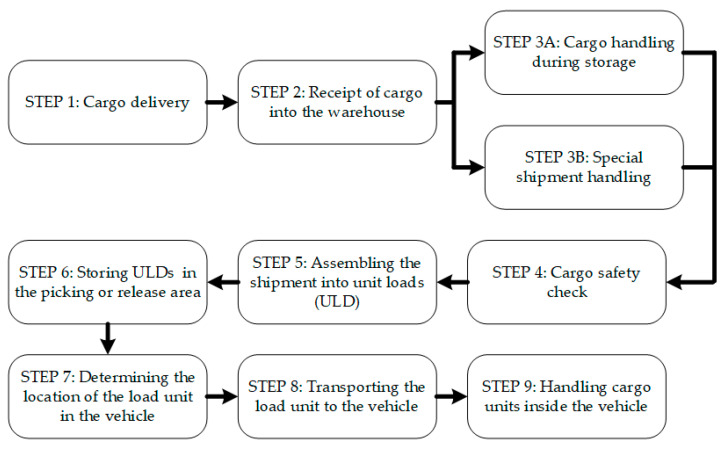
Sub-processes for handling exported shipments.

The study identified two basic cargo handling processes carried out in the CARGO terminal area: (1) the process of handling imported air shipments and (2) the process of handling exported air shipments. Both of these processes were distinguished: eight logistics sub-processes for handling imported shipments (Figure 3) and nine sub-processes for handling exported shipments (Figure 4), which were described at the level of handling operations. Due to the formulated purpose of the article and the level of detail required in further research steps, the presented results do not present detailed process maps at the operational level but only present diagrams showing the order of the distinguished logistics sub-processes implemented within the two distinguished core processes. However, in presenting the results obtained in further research steps, references will appear already at the operational level.

The analysis also made it possible to distinguish the primary actors involved in handling processes in the warehouse zone and to identify the roles assigned to them that are performed in the processes. These include the following:

- Shipper—a shipping company or direct shipper (e.g., a manufacturing company or an individual) responsible for handling the shipment in terms of booking a seat on the aircraft;
- Cargo agent—a person with the authority to handle shipments: WHA and/or LAR and/or DGR in category six and handling the respective internal transport and with authority to handle goods after security screening;
- Operator—a person with the authorization to handle the given internal transport and the mandatory Basic Cargo authorization and possible additional WHA and/or DGR category seven and/or eight authorizations to handle consignments after security control;
- Security Control employee—a person with DGR authorization in category 12 and to operate the Heimann X-ray viewer;
- Customs and Revenue employee—a person with authorization for customs and revenue handling of imported and exported shipments.

Based on Basic Cargo’s training documentation, the documents applicable to air cargo handling were defined. The air waybill (AWB), Cargo Manifest, Cargo Damage Report—CDR, and other documents accompanying air cargo handling were analyzed. At the same time, the general conditions for acceptance of goods and the applicable rules for the classification of cargo according to the IATA guidelines were defined. In accordance with them, air transport distinguishes shipments of, among others, general cargo, AVI—Live Animals, DG—Dangerous goods, HUM—Human Remains, PER—Perishable Cargo, TCR—Temperature Control, DIP—Diplomatic, and VAL—Valuable cargo. These shipments are specially marked with graphic symbols and subject to special packaging procedures, which are inspected in detail before the shipment is released for export. In particular, this applies to hazardous materials, which are marked with additional symbols and letter markings, the identification of which should be assisted by AR technology. Classification of shipment types is important from the point of view of the handling procedures implemented, which will have to be mapped in the augmented reality system.

Guidelines have also been developed for labeling and information that should be placed on cargo packages. These are critical elements for the designed AR system, which these markings must identify to support the operator.

### 4.2. Stage 2—Characterization of Current Information Flows

All information flows were identified according to the knowledge base structure presented in Section 2. A spreadsheet was used to record the data, which allowed quick analysis and grouping of the collected characteristics. A separate spreadsheet was created for each of the identified sub-processes. Sample characteristics for the selected process are shown in Table 1.

Analysis of the collected data made it possible to identify current information gaps that may be the source of adverse events. The results also made it possible to assess the efficiency and resilience of the process to the disruptions occurring. The potential for improvement and opportunities for automation of selected operations were identified on this basis.

### 4.3. Stage 3—Visualization of the Physical Warehouse

**Figure 5:**
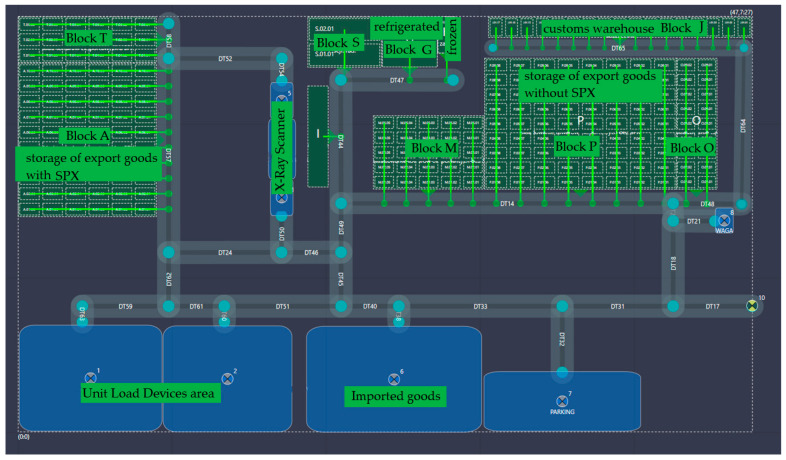
Visualization of the warehouse.

Visualization of the warehouse means a digital representation of its volume (length, width, and height) but also the adopted scheme of organizational flows, considering the people involved, the equipment, and the specifics of the cargo handled. The visualization of the studied object is shown in Figure 5.

The visualization of the investigated warehouse includes ten storage zones, which have been designated as blocks in the system, a collection of adjacent storage locations. Each block in the system has its characteristics, including (a) the number/name of the block; (b) the number of rack spaces in the X dimension (rack depth); (c) the number of rack spaces in the Y dimension (block width); and (d) the number of rack spaces in the Z dimension (block height). Designated storage zones refer to

- storage of export goods without SPX (Block M, Block O, and Block P);
- storage of export goods with SPX (Block A and Block T);
- refrigerated goods warehouse (Block G);
- frozen goods warehouse (Block H);
- customs warehouse (Block J);
- storage of radioactive goods and valuable shipments (Block S).

To serve the designed warehouse, 30 transport routes have been defined. Each road has its characteristics in the system, including (a) name/number of the transport road; (b) X and Y coordinates for marking the beginning and end of the road; (c) numbers of blocks and storage areas accessible from the road; and (d) numbers of other transport roads accessible from the road.

In addition, within the warehouse, the zones for construction of air pallets, preparation and unloading of air pallets, security check station, security check end locations, security check start location, import storage, parking, scale, and warehouse gate were designated. Points identified in these zones were noted with X and Y coordinates.

### 4.4. Stage 4—Preparation of the Scope of Automation of Handling Operations Based on the Functionality of the Information System

Logistics operators use various ERP and WMS systems. Therefore, in determining the potential opportunities for automation, the research team was guided by best practices in various warehouse systems and the specifics of cargo handled in air transportation. At the same time, the potential to automate information flows was validated by the functionality of available IT solutions, the analysis of which was carried out in preparation for the study.

The design of the functionality of the information system supporting cargo flow management was considered separately for export and import shipments. The scope of the automation of handling operations for export shipments concerned the analysis of six logistics processes related to handling such cargo, while five processes were analyzed for import shipments. For each process, operations were identified where automation of information flow was proposed. For each proposal, the expected effects of improving process execution were indicated. Table 2 shows the proposed scope of automation for the process “Receipt of cargo to the warehouse”. Analogous studies were prepared for the other processes.

As a result of the analysis, it was also possible to identify currently impossible operations to automate for the warehouse under study. An example of such an operation in the described process of receiving cargo to the warehouse was entering comments and signing the CMR document. However, it should be noted that such results of the analysis do not exclude the possibility of introducing automatic solutions in the future. Their introduction requires one in many cases, procedural changes (often resulting from existing regulations), as well as costly infrastructure changes.

### 4.5. Stage 5—Preparation of the Functionality Design of the AR System

The functionality design of the AR system was developed based on the results of the process analysis, the scope of implementation of automation of handling operations, and the analysis of the functionality of AR tools available on the market. For each operation indicated to be handled using AR technology, the scope of support, the characteristics of the support, and the form of communication between the operator and the glasses were defined. Table 3 presents a summary of all the operations supported by the AR tool.

## 5. Discussion

The results presented in Section 4 allow us to conclude that the article’s aim has been achieved. Based on the analyses and observations, the air cargo handling process at the CARGO terminal was characterized. On this basis, the specifics of the handling operations and the conditions under which they are carried out were identified. This made it possible to identify those parts of the process that generate a need for information support, often in real time, and for which the introduction of an AR system would be justified. Among the most important issues concerning the specifics of air cargo handling processes are the following:

- the lack of fixed storage locations, causing difficulties in the distribution and picking of cargo in a short period;
- the large number of variables determining the location of cargo in the warehouse—(a) the timing of cargo release for transport; (b) the location of cargo from other sources that will be placed in common transport packages; (c) the weight, size, and type of cargo;
- the wide range of markings used for goods transported by air.

Considering the specificities of air cargo handling in the CARGO zone identified in this way, a functionality was proposed for the AR system that would support the execution of selected operations in the five defined phases of warehouse handling.

First of all, it should be emphasized that the results obtained in the research indicate a high demand for the automation of information flows and a high potential for AR technology to support warehouse service personnel. The process analysis and the identified need for real-time information delivery made it possible to identify critical operations whose support in the form of automation of collection, sharing, and processing will significantly affect the effectiveness and efficiency of the implementation of the entire process. Operations related to identifying air cargo were considered critical, particularly aspects related to recognizing markings for special shipments, which require an appropriate handling procedure. Therefore, it is necessary to develop a system to support the verification of the correctness of air cargo markings. This system should have the following functionality:

- detection of markings applied to a given shipment or baggage;
- indication of missing markings;
- identification of irregularities in applied markings (e.g., incorrect orientation, mislabeling, soiling, and damage).

The second critical operation is to identify and guide the operator to the correct location where the cargo should be stored. The distribution of cargo in warehouses handling air cargo is an NP-hard problem due to the complexity and multidimensional nature of the processes involved. The process analysis indicated that the optimization of cargo distribution in the storage area cannot be based on traditional methods described in the literature due to the specificity of cargo flows in air cargo handling. Specific handling conditions determine all the logistical processes related to air cargo deployment, storage, picking, and loading. Important factors determining these processes include the following:

- varying handling requirements resulting from, among other things, the physical characteristics of shipments (e.g., maximum pressures), but also specific customer requirements;
- heavily heterogeneous cargoes that make it difficult to group and distribute cargoes in a shared space—including irregularity of shape and the need to ensure isolation of cargoes from other cargo groups;
- organizational impediments—taking into account the priority of the shipment due to the timing of transport and the need to load several shipments on one ULD (e.g., described by one AWB or sent to one intermediate or final destination).

At the same time, the loading stage was considered to be a critical process for handling air shipments. This is because, in the case of air transport, there is the problem of optimizing loading in a limited space, in which a known set of heterogeneous cargo units must be loaded into a known number of (usually) heterogeneous containers (available ULDs) under additional constraints arising from the specifics of air transport and with the most uniform loading of all ULDs. Therefore, considering the identified air-transport-specific requirements, developing a hybrid algorithm to optimize cargo flows in the service area is necessary. Both of the above scopes of required research will be the subject of further analysis by the project team. These studies complement the AR system under development but are critical to optimizing the handling process and will primarily provide the developed solution functionality that reflects the specifics of air cargo handling at the CARGO terminal.

Preliminary tests conducted to assist warehouse operators with smart AR glasses confirm the results of studies reported in the literature (e.g., in [21,25,26,29,30]). Workers with little experience get to the designated location faster and make fewer mistakes when handling cargo. Above all, the operation of verifying a special shipment is shortened, which also has a positive effect on the safety of the operations undertaken. The accompanying observations and interviews with test participants also identified potential risks and limitations to using ARSG. They also align with the limitations described in the literature (e.g., in [15,16,28,29]) and concern technical, organizational, and ergonomic aspects. In particular, emerging fatigue in operators using ARSGs and the operator’s limited field of vision with AR glasses are significant risks. The battery life with which ARSGs are equipped also proved to be a significant barrier. On the other hand, the formulated challenge for the tool under development is developing a suitable visualization method to ensure the readability of the messages delivered and enable smooth tracking of the operations performed.

## 6. Conclusions

The results presented in the article answer the research questions posed in Section 3. The process analysis made it possible to determine the sequence and scope of handling operations and define the specific conditions for air cargo logistics handling. Thanks to the created map of information flows, the currently existing information gaps in the studied processes were determined, and, above all, the scope of the required automation of information flows supporting the work of operators in the warehouse was formulated. At the same time, the potential for applying AR technology as a tool to support air cargo handling in individual logistics procedures was defined.

The limitation of the presented results is the focus of attention on the studied physical system, taking into account the specifics of the selected air terminal. The authors tried to include a broader view in their analysis by verifying the obtained results with experts employed at other air cargo terminals, but the identified information gaps and potential for automation were formulated based on the evaluation of this specific case. Therefore, future research needs to verify the assumptions made for other real-world facilities where logistics processes may be more complex and diverse.

The results presented provide knowledge for the scientific and business community. From a scientific point of view, the article provides knowledge regarding the specifics of handling cargo flows in air cargo warehouse operations and the challenges of considering them in the developed AR solutions to be applied to the described system. For industry representatives, the results regarding the formulated design assumptions and the scope of possible application of AR tools in the ground handling of air cargo may be of interest. Preliminary results were also presented as part of the discussion, confirming a reduction in the time taken by operations and eliminating operator errors. These arguments may encourage industry representatives to implement augmented reality in their logistics systems. Attention was also drawn to the risks and limitations involved. This information may be relevant for managers formulating functional assumptions for the AR tool under development.

## Figures and Tables

**Table 1:** Knowledge base about information flows accompanying logistics operations in the CARGO terminal.

PROCESS: DELIVERY OF THE SHIPMENT TO THE WAREHOUSE			
Operation: Verification of shipment data delivered to the warehouse			
Person performing the operation (current form): Cargo agent			
Information supporting the implementation of operations	The current form of obtaining supporting information	Information generated after the operation is completed	The current form of the output information generated
details of the person transporting the shipment/driver	sent e-mail from the sender—no standard form	confirmation of data compliance/determination of inconsistency	verbal message given to the operator along with the order to unload the shipment/telephone contact with the sender of the shipment
registration number of the long-distance transport vehicle	CMR (road transport document) or other consignment note		
shipper			
number of loading units in the shipment			

**Table 2:** Proposed scope of automation for the process “Receipt of cargo to the warehouse”.

Operation	Scope of Automation	Justification
Checking whether the vehicle’s seal has not been broken	Automatic ticket generation when a seal violation is detected	Ensuring registration of information on verification processes carried out in the system. Possibility to archive information about detected nonconformities and actions taken.
Checking for damage in each load unit	Identification of damaged load units in the process—e.g., an automatically printed sticker with a local shipment number confirming its damage	Ensuring registration of information on verification processes carried out in the system. Possibility to archive information about detected nonconformities and actions taken
Measurement of the maximum length, width, height, and weight of each accepted load unit and verification of shipment parameters.	Automatic ticket generation with detected damages (connection to the sender’s key)	Registration of order status. Possibility to archive information about detected inconsistencies. Tracking the history of changes.
Preparation of the admission protocol	Assigning data to the shipment_id in the system and automatic redirection to verification of parameters	The measurement results of the unit after entering the system are the basis for verifying the data contained in the notification. The data are used to indicate the location of the warehouse.
Checking whether the vehicle’s seal has not been broken	Automatic ticket generation in case of non-compliance	Registration of order status. Possibility to archive information about detected inconsistencies. Tracking the history of changes.
Checking for damage in each load unit	Automatic generation of the admission report	The cargo agent’s acceptance of the entered and verified data in the system results in the automatic generation of an acceptance protocol

**Table 3:** Operations supported by the AR tool.

Operation	Scope of Support	Description	Message Proposal
PROCESS: DELIVERY OF THE SHIPMENT TO THE WAREHOUSE			
Verification of shipment data delivered to the warehouse	Displaying to the operator a message about an order to unload a shipment with the specified CMR or AWB, along with an indication of the place of unloading the goods	Message about an unloading order should appear in an additional panel (e.g., in the form of an upper bar with icons), along with the date and time of receipt, status, and place of unloading. After selecting the order, navigation to the unloading place should be started.	Graphic form to signal a new order (icon notifying about a new order). Navigation (e.g., showing the way with arrows)
PROCESS: RECEPTION OF LOADS INTO THE WAREHOUSE			
Checking whether the vehicle’s seal has not been broken	Reporting the need to check the seal	Message about the next step in the service procedure	Text message: “Check the seal”. Graphic icon indicating task completion (e.g., thumbs up)
Checking for damage in each load unit	Registration of information about damaged cargo. Possibility to take a photo of the damage.	Message about the next step in the service procedure. Function of taking a photo of the damage using AR glasses and sending it to the system	Text message: “Damaged?” with the option to select “YES/NO”. A graphic form of the “take a photo of the damage” message after selecting the YES option
Measurement of the maximum length, width, height, and weight of each accepted load unit and verification of shipment parameters.	Reporting the need to perform a measurement	Message about the next step in the service procedure. Delivering cargo to measuring devices.	A graphic icon indicating the need for measurement. Navigate with arrows. A graphic icon indicating that the task has been completed
Preparation of the admission protocol	Preparation of the admission protocol	Message about the generated protocol with the option to confirm or report an error	Graphic icons: Green for acceptance and red for rejection (in case of rejection, you must indicate the protocol field that requires change)
PROCESS: STORAGE			
Determining the location and transport of the load to the storage area	Indicating the storage area and leading to the indicated location	Message with the designation of the storage area. Delivery to the indicated location.	Text message: “Transport to” + storage box symbol. Navigate with arrows.
Special shipment verification	Verification of graphic markings on the shipment (e.g., checking whether the shipment contains all required markings)	Message about the required graphic markings on the shipment in accordance with its specifications.	Graphic icons: green—complete marking; red—incomplete
PROCESS: SAFETY CONTROL			
Transport of the shipment to the X-ray machine	Identification of a free X-ray machine and delivery to its location	Message informing about the need to perform a security check. Delivery to the indicated location.	Graphic icon of an X-ray machine. Navigate with arrows.
Determining the location and transport of the load to the storage area	Indication of the storage area and delivery to the indicated location	Message with the designation of the storage area. Delivery to the indicated location.	Text message: “Transport to” + storage box symbol. Navigate with arrows.
PROCESS: PICKING			
Printout of the picking list	Display of items from the picking list with status	The system should include a module of picking lists with their implementation status (e.g., to be implemented, in progress, or completed) and priority. For each list, there should be an “Execute” option, which will open the given list	The list is presented in text form. Graphical icon for “Execute”
Collection of empty ULDs and their transport	Indication of the need for ULD	Displaying the number of ULDs needed to be downloaded (continuously updated)	Text message: “Download X ULDs”
Transport of shipments to the picking zone	Indicating the storage area and leading to the indicated location	Message with the designation of the storage area. Delivery to the indicated location.	Text message: “Transport to” + storage box symbol. Navigate with arrows.
Placing the shipment on ULD	Indicating the location of the shipment on the ULD	The place of storage is indicated by the displayed solid outline. Updating the picking list after the shipment has been placed in the indicated place.	Graphic icon indicating task completion (e.g., thumbs up)
ULD protection	Indication of required security measures for ULD	Display a reminder to apply security measures. The system should include requirements regarding the security measures used for particular types of cargo.	List of requirements presented in text form. A graphic icon symbolizing a security check
Storage of completed ULDs	Indication of the storage area and delivery to the selected location	Message with the designation of the storage area. Delivery to the indicated location.	Text message: “Transport to” + storage box symbol. Navigate with arrows.
PROCESS: RELEASE FOR TRANSPORT			
Transporting the ULD to the vehicle	Delivery to vehicle	Message of release for transport. Support for delivery to the vehicle parking location	Graphic icon indicating release for transport. Navigation by arrows.
Placement and securing of ULDs inside the vehicle	Support for correct ULD stowage and verification of correct cargo securing	Message about the ULD stowage location and verification of correct stowage, displaying a message about the need to secure the cargo	Indicating the location of stowage with the help of the outline of the lump. Text message: “Check ULD security”. Graphic icon indicating the completion of the task

## Data Availability

Data are contained within the article.
